# Predicting Housing Related Delayed Discharge from Mental Health Inpatient Units: A Case Control Study

**DOI:** 10.1007/s10488-022-01209-y

**Published:** 2022-07-21

**Authors:** Anne Honey, Karen Arblaster, Jenny Nguyen, Robert Heard

**Affiliations:** 1 School of Health Sciences, University of Sydney, Level 7, Susan Wakil Health Building (D18), Camperdown, NSW 2006 Australia; 2 Nepean Blue Mountains Local Health District, 2-6 Station St, Penrith, NSW 2751 Australia

**Keywords:** Patient discharge, Delayed discharge, Inpatient mental health, Housing, Alternate level of care

## Abstract

The aims of this study were to identify factors that a) predict whether people experience housing related discharge delay (HRDD) from a mental health inpatient unit; and b) predict the length of HRDD for people affected. By identifying the groups most affected by HRDD, clinicians and policy makers can prioritise and address barriers to timely discharge at both an individual and systemic level. A case control study using a detailed medical record review was conducted in one Australian mental health service. Demographic, clinical, contextual and systemic variables were collected for patients with HRDD in one calendar year (*n* = 55) and a random comparison sample (*n* = 55). Logistical and multiple regression analyses were conducted to identify variables that predict HRDD and length of HRDD. A model that correctly predicted 92% of HRDD and 78% of non-HRDD cases using five variables was developed. These variables were: diagnosis of schizophrenia or other psychotic disorder, physical comorbidity, having a history of violence or aggressive behaviour, being employed and being involved as a defendant in the justice system. The first three variables increased the likelihood of HRDD, while the second two reduced the likelihood of HRDD. For people who experienced HRDD, the only variable that predicted length of delay was staff reported difficulty finding appropriate support services. This model can be used to rapidly identify patients who might be at risk of HRDD and commence coordinated actions to secure appropriate housing and supports to facilitate timely discharge, thereby addressing a current practice gap. These findings highlight the intersection between health, housing and disability services in the lives of people with serious mental illness, and the need for a whole of government approach to investment and integration to address systemic barriers to suitable housing and supports.

Many people who are admitted to an acute hospital for psychiatric treatment experience delayed discharge. Delayed discharge occurs when patients remain in hospital after being assessed as clinically ready to leave (Rojas-Garcia et al., 2018). It has been identified as a significant problem internationally, with between 10 and 61% of people admitted to mental health inpatient units affected (Glasby & Lester, 2004; Milner & Impey, 2013). Housing issues are a primary reason, in mental health units, for these delays (Corbluth, 2011), with one Irish study observing that 98% of people with delayed discharge had accommodation related needs (Cowman & Whitty, 2016). We define housing related delayed discharge (HRDD) as when hospital discharge is delayed because the person does not have suitable accommodation to be discharged to. Reasons include: lack of housing options; unaffordable housing; lack of access to community support services to facilitate housing placement and maintenance; and administrative delays relating to social housing or accommodation related funding (Nguyen et al., 2021). Until recently, HRDD from mental health units had not been investigated in Australia. Our recent Australian study, however, found that in one local health district (LHD), while only 3.5% of patients experienced HRDD, the average delay was 78 days (median, 41 days). Based on these findings, HRDD was estimated to cost the Australian health system around $269.5 million per year nationally (Nguyen et al., 2021).

Not only is HRDD likely to be costly, it can cause a plethora of other problems. Firstly, it can prevent the admission of new patients into the unit (Corbluth, 2011; Impey & Milner, 2013; National Institute for Mental Health in England, 2007), preventing access to appropriate care for people experiencing acute mental ill health. It is also thought to cause difficulties for organisations and staff including stress, burnout and compromised interprofessional relationships (Imai et al., 2004). Most importantly, it is detrimental to the people affected. Our qualitative study of the perspectives of people experiencing HRDD from mental health units found that participants reported a pervasive lack of choice and control over ‘the basics in life’, ‘how I spend my time’, ‘who I spend time with’, and ‘my future’, resulting in reduced mental and physical well-being and anticipated difficulty transitioning back into the community (Chua et al., 2022). This lack of choice and control represents an infringement of consumers’ rights under the United Nations Convention on the Rights of Persons with Disabilities (United Nations, 2006), including their rights to: liberty (Article 14); choice of living situation and inclusion in the community (Article 19); and participation in all areas of life (Article 9).

Identifying people at risk of HRDD can assist clinicians and policy makers to address this problem within inpatient mental health units. While no research could be located that investigated predictors of HRDD specifically, a small amount of international research, especially from Canada, has investigated predictors of delayed discharge from mental health units more generally. A wide range of potential contributors have been reported, but findings from these studies vary, likely depending on the different definitions of delay used, populations included, variables tested, and analyses performed. Some studies described the delayed sample only (Poole et al., 2014), provided descriptive statistics for delayed and comparison samples (Butterill et al., 2009), or using bivariate comparisons (Little et al., 2015). Multivariate analyses (Kelly et al., 1998; Little et al., 2019) have found that delayed discharge was predicted by: impairment in activities of daily living, moderate to severe cognitive impairment, older age, being male, having a primary language other than English or French, being unmarried, aggressive behaviours, social isolation, “lack of insight” into mental health, a history of substance abuse (Little et al., 2019), requiring placement in a mental health boarding home and being waitlisted for placement (Kelly et al., 1998).

The international research on predictors of delayed discharge from mental health services cannot necessarily provide an accurate picture of what is happening in an Australian context due to differences in health and social care systems. Further, research has not specifically examined the predictors of HRDD. Better understanding about these issues is needed to inform policy makers and health professionals to enable them to appropriately prioritise this issue and develop strategies to prevent HRDD, especially for the people most at risk.

Hence, the purpose of this study was to begin to address the gaps in the literature by developing parsimonious multivariate models to answer the following research questions in one Australian Local Health District:

1. What factors predict whether people experience HRDD?
2. What factors predict length of HRDD for the people affected?

## Methods

### Study Design

A case control study was conducted using a detailed review of data from individual electronic medical records.

### Context

The Local Health District (LHD) studied has an estimated population of 379,000 people, which encompasses approximately 7.6% of the Sydney population (NBMLHD, 2017). It is a diverse area, including rural, regional and metropolitan areas, is on the outskirts of Sydney and encompasses areas in both the least and most disadvantaged economic groups. NBMLHD has five inpatient mental health units: four at a tertiary referral hospital and one at a peripheral regional hospital, with a total of 85 beds. It is against hospital policy to discharge a patient into homelessness, so until accommodation can be secured, a patient will remain in hospital. Our previous study found that HRDD accounted for 11.6% of all bed days in a calendar year in this LHD (Nguyen et al., 2021). However, data on HRDD are not routinely collected in Australia, making this a relatively hidden problem.

According to a recent international report, Sydney has the second least affordable housing in the world, surpassed only by Hong Kong. (Cox, 2022). Anglicare’s annual snapshot found that in 2022, of 14,522 properties available for private rent, only 8 were affordable for a person living on a Disability Support Pension (Anglicare 2022). While Australia provides social housing, waiting times are often greater than ten years, with 55,000 people being on the wait list at 30 June, 2021 (Department of Communities and Justice, 2021). These market issues likely contribute to HRDD.

The study was conducted during implementation in the LHD of the National Disability Insurance Scheme (NDIS). This federal government program provides individualised support, including for people designated as experiencing psychosocial disability (Parliament of Australia, 2016). It is intended to support people with various needs, including housing. While it does not provide housing, it does provide funding for supports, including for people living in social or disability-specific housing. While the NDIS provides funding for people who are eligible for individual support packages from market providers, the concurrent withdrawal of block funding from other services has left some people without support (Mavromaras et al., 2018). Further, discharge staff report that the complex and slow process of approval for funding can contribute to HRDD (Nguyen et al., 2021).

### Sample

The sample of people who experienced HRDD consisted of working age people (15–64 years) who were discharged from NBMLHD mental health units in 2018 and whose discharge was delayed due to housing related factors. We restricted our sample to people under 65 years of age because patients above working age tend to show different patterns and causes of delay (Impey & Milner, 2013; Lewis & Glasby, 2006; Poole et al., 2014). Because delayed discharge is not recorded centrally in the LHD, the identification of these patients required several strategies. Occupational therapists and social workers in the mental health inpatient units were asked to maintain a spreadsheet of patients identified as ‘ready for discharge, awaiting housing’ during 2018. These records, however, were incomplete due to staffing issues throughout the year. Therefore, the length of stay reports produced by the discharge planner, and the records of complex care discharge planning meetings were examined to identify other potentially eligible patients. Ninety-five people were identified as experiencing possible HRDD during 2018. Upon review of the Electronic Medical Records (eMR), 36 people were excluded from the sample because they were outside the age range (n = 13), did not experience HRDD (n = 23) or were not discharged in 2018 (n = 4). This resulted in a final sample of 55 people discharged in 2018 who experienced HRDD.

A comparison sample of patients who did not experience HRDD was obtained from a report of routinely collected data on all patients discharged from the five inpatient mental health units during 2018. This report included data from 1594 people in the target age group (15–64 years). In multivariate models predicting group membership, parameter estimates are most accurate if group sizes are equal (Hosmer et al., 2013, p.403). Due to the mismatch in group sizes between people who did and did not experience HRDD (55 versus 1539), a random sample of 55 non-HRDD people was generated by SPSS to provide an sample of equal size, and extracted for use in the analysis.

### Data Collection

A detailed review was conducted of patient data, progress notes and meeting notes in the Electronic Medical Record (eMR) for the selected people. Demographic, clinical, contextual and systemic variables were collected based on the factors that previous research and clinical experience suggested might be associated with or contribute to HRDD (e.g., Nguyen et al., 2021), or that were indicated from the notes themselves as causing challenges for discharge. These included factors that were identifiable at admission (such as age), issues that occurred throughout the admission (such as encountering delays with obtaining funding through the National Disability Insurance Scheme (NDIS)), and indicators noted on discharge which had potential to delay discharge (such as being treated in the community under a legal order). For people experiencing HRDD, the length of delay was calculated by comparing the date of designation as “ready for discharge” and the actual discharge date. Where records were complex or unclear, two researchers reviewed the files to ensure accuracy of data collection.

### Data Analysis

In order to identify variables associated with HRDD (aim 1), and the length of delay for patients who were delayed (aim 2), potential predictor variables were screened in two steps. First, any categorical variable with a category holding less than 10% of cases was excluded from further analysis. Second, Spearman correlations, Phi coefficients, nonparametric point biserial correlations or Eta coefficients were calculated, as appropriate, between each remaining potential predictor and the dependent variable: HRDD (Yes/No) for research question 1, and logarithm base 10 days of delay for research question 2. Following Hosmer et al., (2013, p.91), potential predictors with a bivariate p < 0.2 were chosen for inclusion in the initial multivariable models.

Because of the relatively low number of cases, there was a risk of having too many variables in an initial model. Therefore, for both research questions, modelling proceeded in three stages. In the first stage, variables were classified into categories as demographic, clinical/contextual, or systemic. These sets were analysed as separate initial models. For research question 1, initial models were produced for all three categories, but for research question 2, only two initial models were needed. In the second stage of modelling, significant predictors in the initial models were used to make a combined model for each research question. In the third stage. predictors which were significant in the two combined models were used in final reduced models. Multivariable modelling used multiple logistic regression for research question 1 and multiple linear regression for research question 2. All data analyses were carried out using SPSS version 26.

### Compliance with Ethical Standards

Ethical approval was obtained from the Nepean Blue Mountains Local Health District (NBMLHD) Human Research Ethics Committee. The study was conducted in accordance with ethical standards laid down in the Helsinki Declaration and the National Statement on Ethical Conduct in Human Research (National Health and Medical Research Council, Australian Research Council, & Universities Australia, 2018). As the study was considered low risk, privacy was protected, and patients may be uncontactable, informed consent was waived. At the outset of the study consultation occurred with the consumer and carer advisory body for the mental health service and this informed the focus of the project. The Authors declare no conflict of interest.

## Results

### HRDD and Comparison Samples

Both the HRDD group and the comparison sample of people who did not experience HRDD consisted of 55 people. The HRDD group had spent significantly more days in hospital during 2018 (mean = 78 days) than the comparison group (mean = 9 days). The two samples are further described in Table 1.

**Table 1:** Description of HRDD and comparison samples

Variable	People experiencing HRDD (n = 55)	People not experiencing HRDD (n = 55)
Demographic variables		
Gender		
Female	24	28
Male	31	27
Age		
Range	16–61	15–63
Mean	36	34
Country of birth		
Australia	50	44
Overseas	5	11
Aboriginal or Torres Strait Islander status		
Yes	3	8
No	52	46
Primary language spoken at home		
English	55	52
Other	0	3
Marital status		
Partnered	4	11
Unpartnered	48	42
Unclear	3	2
Employment at admission		
Employed	1	22
Unemployed	50	27
Unknown	4	6
Primary source of income		
None	6	6
Family	0	6
Job	0	17
Disability support pension	36	7
Other government benefit	9	11
Other	0	4
Housing stability on admission		
Stable	29	37
Precarious	7	14
Homeless	19	4
Housing stability at discharge		
Stable	27	42
Precarious	23	13
Unknown	5	0
Legal status during admission		
Voluntary	18	29
Involuntary	36	24
Mixed	1	2
Clinical and contextual variables		
Number of mental health diagnoses		
Range	1–5	1–4
Mean	1.8	1.6
Diagnoses		
Schizophrenia **s**pectrum/other psychotic disorders	41	19
Bipolar and related disorders	6	9
Depressive disorders	12	17
Anxiety disorders	6	10
Trauma and stressor-related disorders	8	15
Substance-related and Addictive disorders	9	5
Personality disorders	12	8
Other psychiatric diagnosis	3	4
Comorbidity		
Neurodevelopmental disorders	10	6
Dementia/cognitive impairment	5	0
Physical disability/health condition	14	1
Historical and social issues		
Fire starting/property destruction	6	5
History of criminal justice system involvement (e.g., conviction or gaol time)	9	15
	15	32
Self-harm	32	30
Problematic substance use	35	16
Aggressive behaviour/violence	13	20
Victim of violence	21	22
Significant ongoing family conflict	26	28
Significant social isolation	17	13
Housing on admission do not want return		
Systemic variables		
NDIS status on admission		
Active participant	7	3
Applied/applying	2	0
Not applied	46	52
NDIS status on discharge		
Active participant	12	3
Applied/applying	7	0
Declined	2	0
Not applied	34	52
Issues recorded		
NDIS administrative delay encountered	10	0
Difficulty securing appropriate community support	20	0
services		
Rejection from rehabilitation services	5	0
Discharged with legal order		
Community Treatment Order	15	2
Financial management Order	4	1
Guardianship Order	7	1

### Initial Screening

In step 1 of the initial screening, potential predictor variables were excluded because of low numbers in some categories (fewer than 10% of cases in a category) which could not be resolved by collapsing categories. This criterion led to the removal of a number of potential predictor variables for each research questions (marked with # in Table 2). Step 2 of initial screening was to calculate bivariate associations between remaining potential predictors and 1) housing related delayed discharge (yes/no); 2) log10 days of delay. For HRDD (yes/no), nonparametric point biserial correlations were used for ordinal and ratio variables (e.g., housing stability, age), phi coefficients were used for dichotomous variables (e.g., employed/unemployed) and Cramer’s V coefficients were used for categorical variables (e.g., local government area). For days of delay, Spearman correlations were used for ordinal and ratio variables, nonparametric point biserial correlations were used for dichotomous variables, and Eta coefficients were used to assess categorical variables (Table 2).

**Table 2:** Bivariate correlations between demographic variables and delayed discharge (n = 110)

Variable	Delayed Discharge Yes/No bivariate correlation (p)	Days of Delay [log 10] bivariate correlation (p)
*Demographic variables*		
Gender	0.07 (0.45)	− 0.16 (0.24)
Age	0.11 (0.25)	0.11 (0.41)
Country of birth	− 0.16 (0.11)	#
Aboriginal or Torres Strait Islander status	− 0.16 (0.11*)	#
Primary language spoken at home	#	#
Marital status	0.19 (0.06)*	#
Employed at admission	− 0.51 (< 0.001)*	#
Source of income	#	0.21 (0.36)
Housing stability on admission (homeless, precarious or stable)	− 0.22 (0.02)*	0.09 (0.54)
Housing stability at discharge	0.24 (0.02)*	− 0.25 (0.08)*
Legal status on admission (voluntary vs involuntary/mixed)	− 0.2 (0.03)*	− 0.15 (0.27)
Local government area	0.20 (0.20)*	0.15 (0.75)
*Clinical and contextual variables*		
Number of mental health diagnoses	0.08 (0.40)	− 0.03 (0.82)
Schizophrenia **s**pectrum**/** other psychotic disorders	0.40 (< 0.001)*	0.16 (0.23)
Bipolar and related disorders	− 0.08 (0.41)	− 0.27 (0.049)*
Depressive disorders	− 0.10 (0.28)	0.03 (0.83)
Anxiety disorders	− 0.10 (0.28)	0.02 (0.83)
Trauma and stressor-related disorders	− 0.16 (0.10)*	0.07 (0.60)
Substance-related and Addictive disorders	0.11 (0.26)	− 0.16 (0.24)
Personality disorders	0.09 (0.33)	− 0.08 (0.56)
Other psychiatric diagnosis	#	#
Neurodevelopmental disorders	0.10 (0.28)	0.10 (0.94)
Dementia/cognitive impairment	#	#
Physical disability/health condition	0.34 (< 0.001)*	0.04 (0.79)
Fire starting/property destruction	0.03 (0.75)	0.03 (0.83)
Criminal behaviour (conviction, gaol time)	− 0.13 (0.17)*	-0.20 (0.83)
Self-harm	− 0.31 (0.001)*	0.03 (0.85)
Problematic substance use	0.04 (0.70)	− 0.13 (0.35)
Aggressive behaviour/violence	0.35 (< 0.001)*	− 0.11 (0.44)
Victim of violence	− 0.14 (0.15)*	− 0.22 (0.11)*
Significant ongoing family conflict	− 0.02 (0.85)	− 0.23 (0.09)*
Significant social isolation	− 0.04 (0.71)	− 0.13 (0.33)
Housing on admission do not want return		− 0.17 (0.23)
*Systemic Variables*		
NDIS status on admission	0.18 (0.07)*	0.18 (0.07)*
NDIS discharge status Active/Applied vs Not Applied	0.40 (< 0.001)*	0.36 (0.006)*
Difficulty securing appropriate community support services	0.47 (< 0.001)*	0.31 (0.02)*
Discharged with Community Treatment Order	0.13 (0.17)*	0.29 (0.03)*
Discharged with Financial Management Order	#	#
Discharged with Guardianship Order	#	0.21 (0.12)*
NDIS administrative delay	#	0.26 (0.06)*
Rejection from rehabilitation services	#	#

*Variable meets criteria for inclusion in initial multiple models

^#^Excluded due to low numbers in some categories which could not be resolved by collapsing categories

### Research Question 1: Factors Associated with HRDD – Logistic Regression Modelling

In stage 1 of the modelling, three initial multivariable models were run, using demographic, systemic and behavioural/diagnosis predictors as indicated in the table. NDIS status on admission correlated too highly with NDIS status on discharge for both to appear in one model, so only the former was included in the Systemic model. It should be noted that NDIS administrative delay was one of the variables excluded due to low numbers in some categories as only 10/110 people were recorded as experiencing this issue. However, all 10 of these people were in the HRDD group.

In stage 2 of the modelling, predictors significant in the initial multivariable Demographic, Systemic, and Clinical/Contextual models were included in a Combined model. Predictors in this Combined model were a diagnosis of schizophrenia spectrum or other psychotic disorders, physical comorbidities, aggressive or violent behaviour, being employed at admission, a history of criminal behaviour, and Aboriginal or Torres Strait Islander status.

Aboriginal or Torres Strait Islander status was non-significant in the Combined model and was removed in stage 3 of the modelling to produce a final reduced model (Table 3). All predictors were significant in the final reduced model. It had a Cox & Snell R^2^ = 0.51, Nagelkerke R^2^ = 0.68, and successfully predicted 85% of overall cases. It correctly predicted 78% of non-delayed cases, and 92% of delayed cases.

**Table 3:** Final model of factors which predict housing related delayed discharge

Predictor	Beta	s.e	p	OR (95% CI)
Employed at admission	− 4.82	1.83	0.008	0.008 (0–0.29)
Schizophrenia Spectrum and Other Psychotic Disorders	2.07	0.65	0.001	7.89 (2.21–28.20)
Physical disability or health condition (Yes/No)	3.17	1.21	0.009	23.70 (2.22–253.07)
Criminal justice system involvement (e.g. conviction, gaol time)	− 2.30	0.87	0.008	0.10 (0.018–0.549)
Aggressive behaviour/violence (reported by family/self/staff)	2.33	0.77	0.003	10.26 (2.26–46.66)

The model shows that a diagnosis of schizophrenia spectrum or other psychotic disorders, physical comorbidities, and aggressive or violent behaviour are associated with higher odds of delay, whereas being employed at admission and a history of criminal justice system involvement (as defendant) are associated with lower odds of delay.

### Research Question 2: Factors Associated with Length of Delay – Multiple Regression Analysis

The second research question focussed on factors associated with the length of HRDD. Half the sample (n = 55) were people classified as experiencing HRDD. The outcome variable of interest was days of delay. The median length of delay was 38 days (range 1–483, IQR = 59) and the mean was 59.15 days (sd = 84.45). Days of delay was heavily positively skewed, but the logarithm to the base 10 of days of delay was normally distributed and so was used as the dependent variable in multiple regression analyses.

As with research question 1, the intention was to create three initial models for demographic, systemic, and behaviour/diagnostic potential predictors. Initial screening suggested only one demographic predictor met the initial selection cut-off of bivariate association p < 0.2. Accordingly, two initial models were created, systemic factors, and personal factors (including both demographic and clinical/contextual variables).

In stage 2 of the modelling, predictors significant in the initial multivariable models were included in a Combined model. Predictors in this Combined model were significant ongoing family conflict, a diagnosis of bipolar and related disorders, and difficulty identifying appropriate community support services. Significant ongoing family conflict, and a diagnosis of bipolar and related disorders, were non-significant in the Combined model.

Stage 3 of the multivariable modelling consequently used only one predictor in the final reduced model (Table 4), difficulty identifying appropriate community support services. This model was significant (F1,53 = 6.77, p = 0.012) but with only a modest R^2^ = 0.11.

**Table 4:** Final model of factors predicting length of delay

Predictor	Beta	95% CI for Beta	s.e.	t	p
Difficulty finding appropriate community support services	0.36	0.08–0.64	0.14	2.60	0.012
Constant	1.36	1.20–1.53	0.08	16.39	< 0.001

People experiencing difficulty finding appropriate community support services were likely to experience an increased delay of 1.72 days, on average, compared to people not experiencing such difficulty.

## Discussion

This research has generated a robust model to predict housing related discharge delays, which correctly predicted 92% of HRDD and 78% of non-HRDD cases using a parsimonious set of variables. Diagnosis of schizophrenia or other psychotic disorder, physical comorbidity, and history of violence or aggressive behaviour increased the risk of HRDD. The model indicates that the more of these features that are observed in a patient, the greater the risk that they will experience HRDD. Being employed and being involved with the justice system reduce the likelihood of HRDD. For people who experienced HRDD, the only variable that predicted length of delay in the final model was that staff reported difficulty finding appropriate support services.

While in bivariate analysis a considerable number of variables appeared to predict delay, many of these correlations disappeared in the final multivariate model. This is consistent with a situation where, for people who experience HRDD, multiple pathways can explain the extent of delay. These may include individualised factors such as diagnoses associated with cognitive impairment, legal orders and rejection from rehabilitation services. Alternatively, individualised factors or variables that are not recorded in case notes may be responsible for this finding.

The finding that a diagnosis of schizophrenia or another psychotic disorder predicts HRDD supports previous international studies of delayed discharge (e.g., Butterill et al., 2009; Kelly et al., 1998; Poole et al., 2014), but not the most recent, large, Canadian study that used multivariate modelling (Little et al., 2019). This may be due to differences in other variables available, with the Canadian study including variables such as “insight” into a mental health condition and number of previous hospital admissions, which were unavailable from our data. Little et al.’s study also looked only at discharges that were delayed by more than 30 days, thus representing a group of people experiencing more serious delays, and included all delays, not just those that were housing related. Little et al. suggested that the failure of a diagnosis of psychosis to predict delay may have been related to a recent emphasis and increase in access to services for these people in Canada. Interestingly, having a diagnosis of psychotic disorder was not related in our multivariate model to how long a patient was delayed for.

Other contributors to the final model for HRDD (Yes/No) are variably consistent with the delayed discharge literature. The importance of aggressive behaviour is supported by Little et al. (2019) and Butterill et al. (2009) found that physical illness was more common in people with delayed discharge. Consistent with our findings, Poole et al. (2014) described employment as rare amongst people with delayed discharge, while Kelly et al. (1998) found that employment was not a predictor of delay. In the model produced in this research, people with a history of criminal justice system involvement (as defendant) had lower probability of HRDD. Although this may seem counterintuitive, it reflected the ongoing nature of that involvement for five non-delayed people, whose discharge destination involved gaol or police custody.

Clinician difficulty identifying which consumers admitted to inpatient mental health units are homeless or at risk of homelessness has been reported as a contributing factor to HRDD (Productivity Commission, 2020). Interestingly, the predictors of HRDD in our final model are variables that are apparent on admission, with the exception of having a history of aggressive or violent behaviour, which included behaviour both prior to and during the admission. This information can be used to facilitate early identification of people who may require additional or intensive assistance with securing accommodation. The model produced in this research is therefore an important contribution to research and policy related to this issue.

In this study NDIS related factors did not predict HRDD due to the small number of people who were in the process of applying for NDIS funding. However, it is noteworthy that all 10 of these participants were in the HRDD group. NDIS processes for people with psychosocial disability have been reported as opaque, complex and difficult to navigate (Hamilton et al., 2020; Nguyen et al., 2021). In their recent scoping review Hamilton et al. (2020) identified specific factors that contribute to these difficulties. These include: (1) inflexibility and complexity of assessment and planning processes; (2) the shortage of affordable housing; (3) vertical siloing of systems such as disability, health and housing; and (4) lack of mental health expertise by NDIS staff. This likely contributes to the finding that difficulty arranging suitable community supports predicts length of HRDD.

### HRDD and community supports

Housing is a human right and is important for recovery for people living with mental illness (Productivity Commission, 2020; United Nations, 2006). It has been estimated that in Australia over 2000 mental health inpatients could be discharged if appropriate housing and clinical and social supports were available (Productivity Commission, 2020). This lack of support services has been consistently reported, especially for people who are not involved with the NDIS (Brackertz et al., 2018; Hancock et al., 2019; Nguyen et al., 2021; Smith-Merry et al., 2018). The findings in this study, and the experiences of clinicians in supporting consumers to obtain appropriate housing and supports in our earlier study (Nguyen et al., 2021), highlight the importance of this issue and the urgent need for systemic action. The importance of NDIS reform and extending services which combine housing and support and have been demonstrated to have a range of positive outcomes including reduced hospital admissions (Bruce et al., 2012), is clear.

While the urgent need for more appropriate housing to be made available is clear, government investment in social housing in Australia has decreased in recent years (Australian Institute of Health & Welfare, 2018). The Australian Housing and Urban Research Institute (AHURI), amongst many other reports, has made recommendations for governments to invest more in social housing. It is clear that the lack of sufficient social housing and community support for people living with mental illness places a burden on the public mental health system. Delayed discharge should thus not be viewed as a problem for health ministries; a whole of government approach is needed to address the problem of housing related delayed discharge in Australia. This echoes repeated calls by the Productivity Commission, National Mental Health Commission and Council of Australian Governments for an increase in investment to provide long-term stable housing options for people living with mental illness to improve their mental health and reduce the need for costly mental health inpatient services. International research suggests that other populations may well encounter parallel issues, with multiple barriers being identified around the discharge of unhoused medical patients in Canada, including inadequate access to support services and temporary accommodation, and the risk of falling through the gaps between health and social service systems (Jenkinson et al., 2022).

### Future directions

There are several possible uses of the model produced in this research. As previously noted, the model can be used by clinicians to identify consumers at risk of HRDD at the point of admission. This would enable early referral to social work and occupational therapy for proactive assessment and intervention to minimise anticipated HRDD. It is possible that an algorithm could be built into electronic medical record systems to enable automated reporting that identifies people admitted to inpatient units who have some or all of the risk factors identified in our model. This could be supported by developing a clinical pathway for HRDD that sets out a standard coordinated set of actions for clinicians that include early assessment, physical health screening and coordination of subspecialty consultations, referrals to housing and support providers, NDIS applications where needed and other relevant actions. Future research might explore whether implementation of the model and its possible uses reduce incidence and/or length of HRDD, and whether the model is generalisable to other mental health services in geographically and socioeconomically diverse areas.

Systematic recording of delayed discharge data within health systems, as occurs in some jurisdictions (e.g., Little et al., 2019) is recommended, given the importance of the problem, to facilitate ongoing monitoring and future larger-scale studies of delayed discharge.

Given the reliance on external agencies to improve timely access to housing and reduce the length of HRDD, building partnerships with providers of social housing is important. Social housing providers can advise the types of personal and clinical information that could be collected by clinicians in mental health inpatient units to assist them to facilitate access to housing. This, in turn, might inform developments in the eMR for systematic recording and reporting of HRDD. Building effective partnerships with social housing providers might also enable enhanced pathways from inpatient care to suitable housing to be developed. In NSW Australia the newly released Housing and Mental Health Agreement (NSW Health and Department of Communities and Justice, 2022) offers a partnership framework that might guide these efforts.

### Limitations

There are several limitations to this study. First, while considerable efforts were made to identify all people who experienced HRDD, some participants may have been missed due to inconsistencies in record keeping. Similarly, the information collected from the EMRs may have been incomplete as issues experienced by patients may not be recorded in every case. Lastly, the sample size of 110 people was relatively small, so some real associations may not have been detected. While raw numbers indicated that more Aboriginal and Torres Strait Islander people were in the not-delayed group, low numbers may have contributed to this variable not being included in the final model.

## Conclusion

This study has produced a model that might be used by clinicians to predict which people admitted to mental health inpatient units might be at risk of HRDD and to prioritise coordinated actions to secure appropriate housing and supports to facilitate timely discharge. However, the problem of HRDD from inpatient mental health services appears symptomatic of wider issues relating to the lack of availability of secure, affordable housing and community support, resources that are fundamental to recovery from mental illness. It is imperative that a whole of government approach be taken to address these issues, including overall additional investment in social housing and housing support.

## Declarations

### Standards of Reporting

The study design and reporting were guided by the STROBE checklist for case control studies (https://www.equator-network.org/reporting-guidelines/strobe/).
